# Distinct Kinetics of Memory B-Cell and Plasma-Cell Responses in Peripheral Blood Following a Blood-Stage *Plasmodium chabaudi* Infection in Mice

**DOI:** 10.1371/journal.pone.0015007

**Published:** 2010-11-23

**Authors:** Eunice W. Nduati, Dorothy H. L. Ng, Francis M. Ndungu, Peter Gardner, Britta C. Urban, Jean Langhorne

**Affiliations:** 1 KEMRI/Wellcome Trust Collaborative Research Programme, Centre for Geographical Medicine Research Coast, Kilifi, Kenya; 2 Division of Parasitology, MRC National Institute for Medical Research, London, United Kingdom; 3 Liverpool School of Tropical Medicine, Liverpool, United Kingdom; Université Pierre et Marie Curie, France

## Abstract

B cell and plasma cell responses take place in lymphoid organs, but because of the inaccessibility of these organs, analyses of human responses are largely performed using peripheral blood mononuclear cells (PBMC). To determine whether PBMC are a useful source of memory B cells and plasma cells in malaria, and whether they reflect *Plasmodium-*specific B cell responses in spleen or bone marrow, we have investigated these components of the humoral response in PBMC using a model of *Plasmodium chabaudi* blood-stage infections in C57BL/6 mice. We detected memory B cells, defined as isotype-switched IgD^−^ IgM^−^ CD19^+^ B cells, and low numbers of *Plasmodium chabaudi* Merozoite Surface Protein-1 (MSP1)-specific memory B cells, in PBMC at all time points sampled for up to 90 days following primary or secondary infection. By contrast, we only detected CD138^+^ plasma cells and MSP1-specific antibody-secreting cells within a narrow time frame following primary (days 10 to 25) or secondary (day 10) infection. CD138^+^ plasma cells in PBMC at these times expressed CD19, B220 and MHC class II, suggesting that they were not dislodged bone-marrow long-lived plasma cells, but newly differentiated migratory plasmablasts migrating to the bone marrow; thus reflective of an ongoing or developing immune response. Our data indicates that PBMC can be a useful source for malaria-specific memory B cells and plasma cells, but extrapolation of the results to human malaria infections suggests that timing of sampling, particularly for plasma cells, may be critical. Studies should therefore include multiple sampling points, and at times of infection/immunisation when the B-cell phenotypes of interest are likely to be found in peripheral blood.

## Introduction

The majority of the human cellular immunological studies are performed using peripheral blood mononuclear cells, as blood is, with a few exceptions [1] the only readily accessible source of cells of the innate and acquired immune system. However during and after infections, particularly long-lasting infections such as malaria, a redistribution of lymphocytes can take place where specific lymphocytes become activated and remain in lymphoid organs or migrate to the tissues rather than circulate in peripheral blood. Thus low, or no, specific responses in peripheral blood may not necessarily imply that the host is hypo-responsive. This makes it difficult to interpret human cellular studies. For example, it has been demonstrated that activated antigen-specific T cells are transiently depleted from the circulation at the peak of infection with *P. falciparum* [2]–[6]. However, in *P. chabaudi* infection, specific CD4^+^ T cell responses were detected in peripheral blood mononuclear cells (PBMC) at late time points after the parasitaemia had been cleared [5]. This suggests that T cell responses in peripheral blood may not necessarily be indicators of the immune responses occurring in lymphoid organs, and that timing the sampling of PBMC from infected individuals may be important to catch responsive T cells.

Much less is known about alterations in the distribution of B cell and plasma cell populations following malaria infection. Since B cell and antibody responses are crucial for protective immunity to blood-stage malaria infections [7]–[10], it is important to understand their nature and regulation. Some studies have shown that B cell numbers are altered in the spleens of mice during blood-stage malaria infection [11], and two reports suggest that B cell subset redistribution also occurs in humans [12],[13]. The changes in the composition and distribution of B cells and plasma cells which occur in secondary lymphoid tissues after immunization and infection [14]–[19] may be detected in peripheral blood as memory B cells (MBC) and plasma cells can circulate or migrate between lymphoid compartments during an ongoing humoral response. A recent study has shown that the spleen, but not blood, is a major reservoir for human virus-specific memory B cells [1]. This information is not available for human malaria.

Experimental models may provide an indication of the usefulness of peripheral blood PBMC as a source of B cells and plasma cells in malaria infections. Here, we have used a mouse model of malaria, *Plasmodium chabaudi chabaudi* (AS) in C57BL/6 mice, and flow cytometry and ELISpot assays, to compare B cell and plasma cell responses in PMBC with those in the spleen (where B cells are activated) and bone marrow (BM) (where haematopoesis leading to production of B cells occurs; and where the majority of long-lived plasma cells reside) during acute malaria infection, to determine whether B cell responses observed in peripheral blood reflect those observed in the other organs, and if it reflects a malaria-specific B cell response. We found that memory B cells were present in the blood in low numbers at all time points tested for up to 90 days following infection, and Merozoite Surface Protein 1 (MSP1)-specific memory B cells could be detected by ELISpot at these times. In contrast, plasma cells and MSP1-specific antibody-secreting cells (ASC) were detectable in blood only within a narrow time period, approximately 10 days following infection. These ASC were likely to reflect a developing plasma cell response, as the majority of CD138^+^ cells in the blood at this time had the characteristics of newly differentiated migratory plasmablasts rather than mature long-lived plasma cells that had been dislodged from the bone marrow.

The results from this comparative study suggest that timing of blood sampling following a malaria infection may be crucial for the detection of antigen-specific B cell responses in peripheral blood.

## Materials and Methods

### Ethics Statement

This study was carried out in strict accordance with the UK Animals (Scientific Procedures) Act 1986. This study was approved by the Ethics Committee of the MRC National Institute for Medical Research, and the British Home Office (PPL: 80/2538).

### Mice

Female C57BL/6 mice aged 6–8 weeks were obtained from the specific pathogen free unit at the National Institute for Medical Research (NIMR), London. For experimental purposes, mice were housed conventionally with sterile bedding, food and irradiated water.

### Infection with *P. chabaudi chabaudi* (AS) parasites

A cloned line of *Plasmodium chabaudi. chabaudi* (AS) originally obtained from David Walliker, University of Edinburgh was used in this study. Stabilates were cryopreserved in blood from C57BL/6 mice. To obtain parasites for experimental infection, an aliquot of the stabilate was injected intraperitoneally into donor C57Bl/6 mice. Blood were taken from the donor mice before peak of parasitaemia and experimental mice were infected by injecting 10^5^ infected erythrocytes intraperitoneally (i.p). Parasitaemia was monitored by examination of Giemsa-stained blood films as previously described [10]. After 45 days of the primary infection, some mice were re-challenged i.p. with 10^5^ infected erythrocytes.

### Antibodies and reagents

Antibodies used were CD19-allophycocyanin (APC) and Biotin, CD138-phycoerythrin (PE), IgM-PE and PerCP-Cy5.5, CXCR4-Biotin, CXCR5-Biotin and GL7-fluorescein isothiocyanate (FITC) (BD Pharmingen), IgD-PE and Biotin, MHC class II-Biotin and B220-APC (eBioscience). Biotinylated antibodies were revealed with streptavidin peridinin chlorophyll protein (PerCP) (BD Biosciences) and streptavidin eFluor™ 450 (eBioscience). Anti-Fc receptor and Rat IgG2b'κ FITC, Rat IgG2a'κ PE, Rat IgG2a'κ biotin, Rat (lavian) IgMκ isotype controls were purchased from BD Pharmingen.

### Multi-parametric flow cytometry analysis

Single cell suspensions of spleen, bone marrow from two femurs and heparinised peripheral blood were prepared in complete Iscove's medium containing 10% fetal calf serum (FCS), 100 units/ml of penicillin, 100 µg/ml of streptomycin, 1 mM of L-glutamine, 12 mM of Hepes (all purchased from Sigma, UK) and 5×10^−5^ M of 2-mercaptoethanol (Invitrogen). Erythrocytes were lysed with red cell lysing buffer (Sigma). Lymphocytes were counted and aliquoted at 1×10^6^ cells/well in 96-well V-bottom plates. Surface staining and washing steps were done with FACS buffer (PBS containing 2% FCS, 5 mM EDTA and 0.01% NaN_3_). To prevent non-specific binding of monoclonal antibodies to the Fc receptors, 25 µl/well Fc receptor-block was added to the cells and incubated for 10 min on ice. Cells were then incubated for 20 min at 4°C with antibodies to detect GL7^+^ populations, transitional B cells, memory/isotype-switched B cells and plasma cells. Cells were then washed twice and fixed overnight with 2% paraformaldehyde in PBS. Data were acquired on a FACS Calibur using Cell Quest Pro (Becton Dickenson) and analysed using FlowJo (Treestar Inc.).

### Preparation of recombinant MSP1_21_

Recombinant MSP1 protein (MSP1_21_, aa _4960–5301_) from *P. chabaudi chabaudi AS* was expressed as his-tagged protein in *Pichia pastoris* SMD1168, as previously described [20], and purified by binding to a nickel-nitrilotriacetic acid (Ni-NTA) agarose column (Qiagen, Hilden, Germany) and eluted with 250 mM imidazole.

### ELISpot Assay for Merozoite surface protein-1 (MSP1)-specific plasma cells

MSP1 antigen-specific plasma cells were quantified by direct *ex vivo* ELISpot assay based on their ability to continuously secrete antibody [21]. 96-well Multi-screen HA Nitrocellulose filtration plates (Millipore) were coated with 50 µl of 10 µg/ml recombinant MSP1 diluted in PBS. As a positive control for total IgG secreting cells, some wells on each plate were coated with goat anti-mouse IgG (Caltag). The plates were incubated at 4°C overnight, washed twice in PBS, then blocked with 200 µl complete Iscove's medium for 1 h at room temperature. The plates were then washed twice with PBS and cell suspensions added at the following numbers: 3.5×10^5^, 1.2×10^5^, 3.9×10^4^ and 1.3×10^4^ per well in 100 µl complete Iscove's medium. The plates were incubated at 37°C, 7% CO_2_ for 5 h, then washed twice in PBS and twice with PBS with 0.1% Tween (PBS-T). 100 µl of goat anti-mouse IgG biotin conjugated antibody (Caltag) diluted 1∶1000 in PBS-T containing 1% FCS was added and the plates incubated overnight at 4°C. Plates were washed four times with PBS-T, and 100 µl of 5 µg/ml alkaline phosphatase avidin D diluted in PBS-T containing 1% FCS added and incubated for 1 h in the dark at room temperature, followed by two washes with PBS-T and two washes with PBS. Detection was carried out by adding 100 µl of BCIP/NBT substrate (Vector Laboratories) and incubating in the dark until blue spots appeared. The reaction was stopped by thorough washing with cold tap water and air-dried. Plates were analysed using the ImmunoSpot reader, (CTL).

### ELISpot Assay for MSP1-specific memory B cells

Memory B cells were detected using a modification of the plasma cell ELISpot previously described [21]. In brief, replicates of three-fold dilutions of cell suspensions of spleen, blood and bone marrow were made on flat-bottomed 96-well plates (Costar), in replicates of 22 wells for each dilution, and cultured for 6 days in 200 µl complete Iscove's medium containing 0.4 µg R595 lipopolysaccharide (Alexis Biochemicals), 1×10^6^ irradiated (1,200 rad) naive splenocytes and 20 µl of supernatant from concanavalin A-stimulated splenocytes, prepared as previously described [22]. Cells were harvested and transferred to 96-well Multi-screen HA Nitrocellulose filtration plates and an *ex-vivo* ELISpot assay for MSP1***-***specific plasma cell detection performed as described above. The frequencies of MSP1-specific memory B cells were determined from the zero order term of the Poisson distribution, using least squares method of curve fit. The goodness-of-fit curve was analysed by linear regression where r^2^ values greater than 0.8 were accepted.

### Statistics

Experiments were analysed using the Mann Whitney non-parametric test on Prism 5 software (GraphPad Software Inc.) and p≤0.05 considered significant.

## Results

### Changes in total PBMC, B cells and plasma cells occur in blood during a *Plasmodium chabaudi* infection

**Figure 1:**
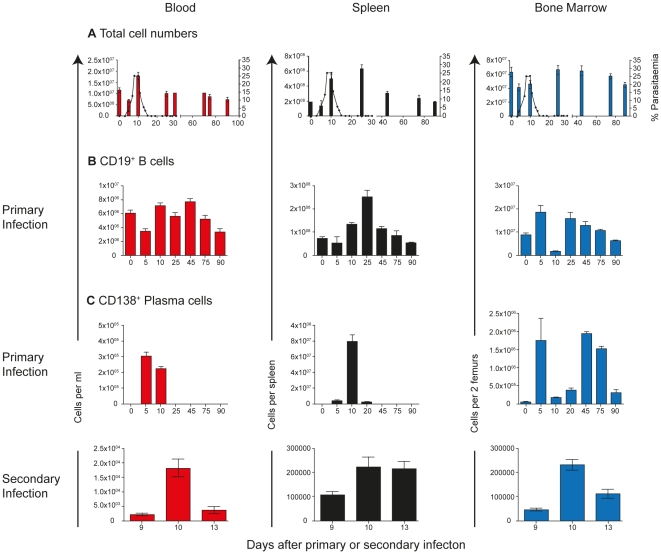
Changes in B-cell and plasma-cell number in different compartments. C57BL/6 mice were injected intraperitoneally with 10^5^ *Plasmodium chabaudi chabaudi* (AS) iRBC and the infection course followed. A) Total PBMC in peripheral blood (red), spleen (black) and bone marrow (two femurs; blue) at different time points following a primary infection; B) CD19^+^ B cells determined by flow cytometry following a primary infection; C) Numbers of CD138^+^ plasma cells/plasmablasts determined by flow cytometry following a primary (upper panel) and secondary infection (lower panel). Gating strategies for CD19^+^ and CD138^+^ cells are shown in Figure S1. The values and error bars shown are the means and the standard errors of the mean (SEM) of 5 to 7 mice.

In order to compare the phenotypes and numbers of malaria-specific B cell- and plasma cells (PC) in PBMC with those in spleen and bone marrow, we first determined the changes in total cellularity in these organs during a *Plasmodium chabaudi* blood-stage infection. Substantial changes in the total cellularity and overall composition of B cells and PC are known to take place in spleen and bone marrow during blood-stage rodent malaria infections [2]–[6], [23]–[25]. In agreement with these studies, we observed a large increase in total cell numbers in the spleen following the peak of parasitaemia, and a transient drop in numbers in the bone marrow just before and at the peak of parasitaemia (Figure 1A). The numbers of PBMC in blood, however, increased transiently only at peak parasitemia and then return to levels of naïve mice by day 20 and for the remainder of the infection (Figure 1A). CD19^+^ B cell numbers in blood did not alter substantially during a primary *P. chabaudi* infection (Figure 1B). By contrast, we observed a 2.5-fold increase in CD19^+^ B cells on day 25 of infection and a depletion of CD19^+^ B cells from the bone marrow at peak parasitaemia. Simlarly, CD138^+^ PC were present only transiently in blood just before and at the peak of parasitaemia (day 5 and 10), and in very low numbers (less than 5% of PBMC; Figure 1C). There was a transient increase in CD138^+^ cells between days 5 and 25 in the spleen during the primary infection. In bone marrow, we observed a bi-modal pattern with a transient peak on Day 5, and a more sustained increase from day 45 onwards. The kinetics of CD138^+^ cells in peripheral blood was similar during a secondary infection. This suggests that timing of blood sampling during the peak of infection, even in individuals who have had multiple exposures, may be critical for detecting malaria-specific PC.

### Transitional, mature naive and memory B cell populations are detected in blood, spleen and bone marrow during a *P. chabaudi* infection

**Figure 2:**
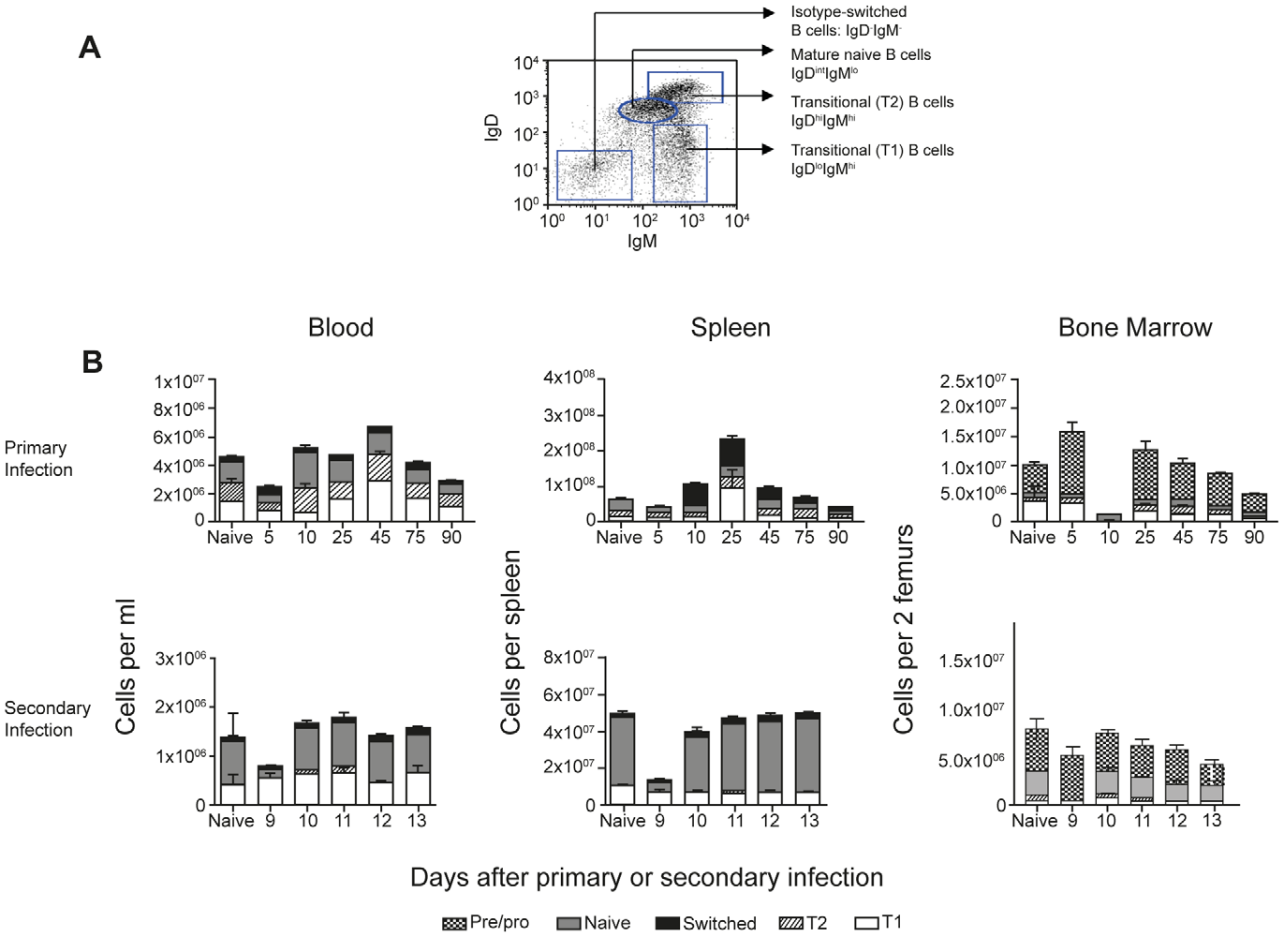
Changes in subpopulations of B cells in different compartments. A) Gating strategy for the identification of transitional T1 (IgM^high^ IgD^low^), T2 (IgD^high^ IgM^high^), naïve B cells (IgD^intermdiate (int)^ IgM^low^) and switched B cells (IgM^−^IgD^−^) on CD19^+^ B cells. B) Numbers of the different subpopulations of B cells were determined by flow cytometry following a primary and secondary infection of *Plasmodium chabaudi chabaudi* (AS). The values and error bars shown are the means and the standard errors of the mean (SEM) of 5 to 7 mice.

Memory B cells are mostly contained within the CD19^+^ IgM^−^ IgD^−^ isotype-switched population of B cells [26]–[28]. To gain an idea of the expected frequency of all “memory” or isotype-switched B cells in peripheral blood during infection compared with spleen, we investigated surface IgM and IgD expression on CD19^+^ B cells by flow cytometry. Based on the relative surface expression of IgM and IgD, transitional, mature naïve and isotype-switched B cells can also be distinguished within the CD19^+^ populations ([29]. Here, IgD^low^ expressing cells are defined as early transitional B cells (T1), although in the spleen this population may also include marginal-zone B cells. IgD^high^ IgM^high^ expressing cells are designated as late transitional B cells (T2), IgD^intermdiate (int)^ IgM^low^ as mature naïve B cells, and IgD^−^ IgM^−^ as isotype-switched B cells, that is, memory or antigen-experienced B cells [29] (as shown in Figure 2A).

During a primary *P. chabaudi* infection, all subpopulations of B cells; T1, T2, mature naïve and isotype-switched were observed in peripheral blood, although their numbers and proportions differed from those in spleen (Figure 2B). Most importantly, the proportion of isotype-switched B cells were only a small proportion of B cells in the blood (less than 10% of B cells, except on day 5 when approximately 20% of B cells had switched), in marked contrast to the spleen, where they comprised 25%-50% of the B cells between days 10 and 45 of infection.

Memory B cells are not thought to reside in the BM [30] and CD19^+^ IgM^−^ IgD^−^ cells in bone marrow are considered to be pro- and pre-B cells [29], [31]–[33]. In naïve mice and for most of the primary *P. chabaudi* infection these cells comprised the largest fraction (>50%) of CD19^+^ cells in BM. Notably, at day 10 of infection, just after peak parasitemia, this immature B cell population was drastically reduced compared with the numbers at day 5 or day 25 (Figure 2B, right panel; Mann Whitney p<0.001). This suggests that immature B cells had been exported from the BM or undergone cell death in large numbers, or that hematopoesis leading to the production of pro- and pre-B cells was severely depressed at the peak of infection.

During a secondary infection with *P. chabaudi*, other than a drop in total numbers, and particularly in mature naive B cell numbers, in all organs at day 9, the relative proportions of the different subpopulations do not appear to alter significantly. Interestingly, with the exception of day 9, the numbers and proportions of all the B cell subpopulations remain very stable during an acute secondary infection, with memory B cells comprising less than 5% of B cells at any time tested. Therefore circulating memory B cells remain at very low frequencies even during a secondary infection.

### GL7 is expressed mainly on immature B cells in the blood during a *P. chabaudi* infection

**Figure 3:**
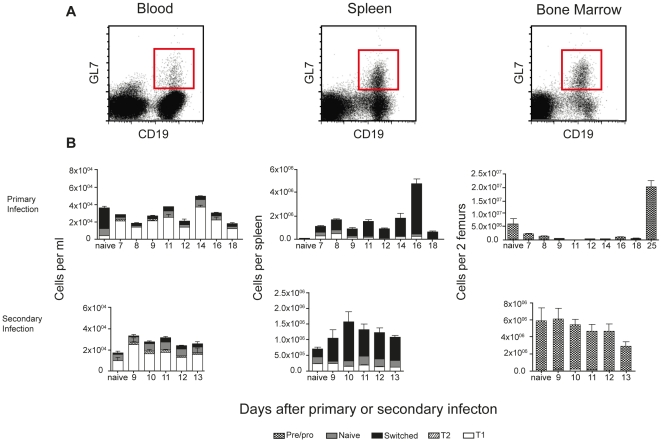
GL7 is expressed mainly on transitional B cells in blood during infection. A) Gating strategy for the identification of GL7^+^ CD19^+^ B cells in peripheral blood (left), spleen (middle) and bone marrow (right). B) The number of CD19^+^ B cells expressing GL7 on different subpopulations; transitional T1 (IgM^high^IgD^low^), T2 (IgD^high^IgM^high^), naïve B cells (IgD^intermdiate (int)^IgM^low^) and isotype-switched B cells (IgM^−^IgD^−^) determined by flow cytometry. The values and error bars shown are the means and the standard errors of the mean (SEM) of 5 to 7 mice.

Another marker used to distinguish antigen-experienced B cells is GL7 [34], [35]. This cell surface protein is expressed at high levels on early pre-B cells, downregulated on mature naive B cells, and then upregulated and highly expressed on germinal centre and post-germinal centre B cells that have undergone affinity maturation and/or isotype switching [35], [36]. Following a primary *P. chabaudi* infection, the proportion and numbers of total GL7^+^ CD19^+^ B cells in PBMC were greater than those of total isotype-switched B cells as described in Figure 2, indicating that GL7 expression on PBMC was not identifying exclusively isotype-switched B cells. Indeed, we found that GL7 was expressed mainly on immature T1 and T2 B cells in PBMC, which had presumably migrated from the bone marrow. In the spleen, GL7 expression was predominantly on isotype-switched B cells (Figure 3B), in agreement with previous studies [34], [37]. In bone marrow, GL7 was expressed on pre-B cells as described previously [34], [37]. Because of the expression of GL7 on immature B cells appearing in peripheral blood during a primary and secondary infection, GL7 cannot be used as a marker for memory B cells in PBMC.

### *MSP1-specific IgG memory B cells can be detected in peripheral blood of infected mice ten days following infection and are maintained for a long time*

**Figure 4:**
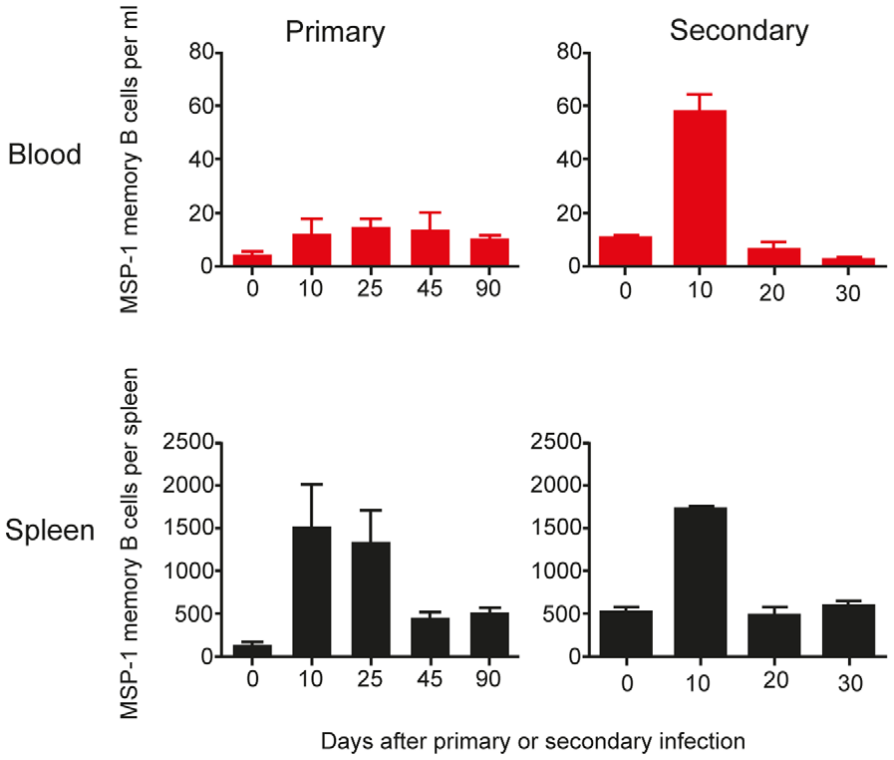
MSP1-specific IgG memory B cells are detectable in peripheral blood in low numbers. The frequencies of MSP1-specific IgG memory B cells were determined by *in vitro* cultured ELISpot assays as described in the experimental procedures. The frequencies of MSP1-specific IgG memory B cells in peripheral blood and spleen following both a primary and secondary infection are presented as memory B cells per ml of blood and per spleen respectively. The values and error bars shown are the means and the standard errors of the mean (SEM) of 5 to 7 mice.

The persistently low frequency of memory B cells in peripheral blood detected by flow cytometry suggested that the frequency of detectable malaria-specific B cells might be very low. We used an ELISpot assay to detect MSP1-specific IgG MBC during primary and secondary infection (Figure 4). In line with the low frequency of isotype-switched MBC, the frequency of MSP1-specific IgG MBC in PBMC as determined by ELISpot was also very low but consistent from day 10 of a primary infection onwards (Figure 4, top left graph, ∼12 MSP1-specific IgG MBC per ml of blood). This was markedly lower than the numbers seen per spleen, which, as described previously [38], peaked at day 10 of infection (figure 4, bottom left graph, approximately 1500 MSP1-specific IgG MBC (per spleen), then contracted (approximately 500 per spleen) and persisted for up to day 90. Thus MBC in peripheral blood can be detected in low numbers after peak parasitaemia, but do not reflect the kinetics of MBC in the spleen.

After a secondary infection, there was a 3.5-fold increase in numbers of MSP1-specific MBC in the spleen at day 10 (Figure 4, bottom right graph, from 500 to 1700 per spleen). A 5-fold increase in MSP1-specific MBC was also observed in peripheral blood on day 10, albeit at a much lower frequency (Figure 4, top right graph, from 15 to 60 per ml of blood) and resuming previous levels by day 20.

MBC could not be detected at significant levels at any time in the BM (data not shown).

These data suggest that the secondary MSP1-specific MBC response in the spleen has taken place very early following re-infection, and by day 20 these cells have most likely died or have differentiated into short-lived plasma cells. Although there was no increase of MBC at day 20 in the blood or bone marrow, it is possible that the MBC have been redistributed to other tissues or lymphoid organs. In blood, whilst frequencies of MSP1-specific MBC remained low throughout primary infection, there was a significant increase in MSP1-specific MBC in blood at the peak of a secondary infection, perhaps reflecting the migration of some MSP1-specific MBC out of the spleen.

### MSP1-specific IgG antibody-secreting cells can be detected in peripheral blood within a narrow window of time post-infection

The transient appearance of plasma cells detected at the peak of parasitaemia by flow cytometry could reflect a *Plasmodium*-specific plasma cell response. As described above for MBC, we could not determine the fraction of MSP1-specific plasma cells/plasmablasts by this method and therefore used an MSP1-specific ELISpot assay to enumerate MSP1-specific IgG antibody-secreting cells (ASC) (plasma cells and plasmablasts).

**Figure 5:**
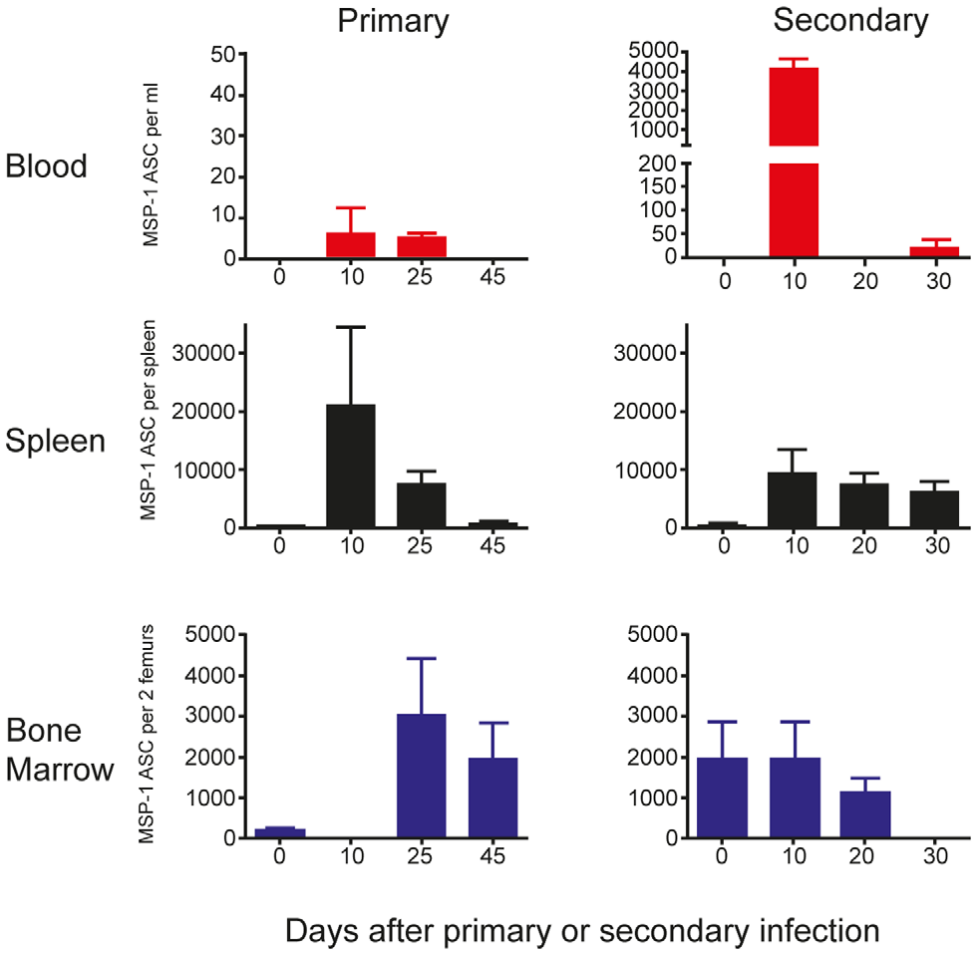
MSP1-specific IgG antibody-secreting cells (ASC) appear transiently in peripheral blood during infection. The frequencies of ASC were determined by direct ELISpot assays as described in the experimental procedures. The numbers of MSP1-specific IgG ASC in peripheral blood, spleen and bone marrow following both a primary and secondary infection are presented as ASC per ml of blood, per spleen and per two femurs respectively. The values and error bars shown are the means and the standard errors of the mean (SEM) of 5 to 7 mice.

The numbers of MSP1-specific IgG ASCs in peripheral blood following a primary infection could only be detected between days 10 and 25 and were extremely low (Figure 5 left graph, top panel, approximately 5 per ml) compared with either spleen (2×10^4^) or BM (3×10^3^) (Figure 5, middle and bottom panels). However, there was a transient but large increase of MSP1-specific IgG-ASCs in peripheral blood (Figure 5, right graph, top panel, approximately 4000 per ml) on day 10 following a secondary infection.

In spleen, maximum numbers of MSP1-specific IgG ASCs were observed at day 10 of the primary infection (Figure 5, left graph, middle panel) and reducing to barely detectable levels by day 45. After a secondary infection, there was a rapid increase in the numbers of MSP1-specific IgG ASCs numbers at day 10, which persisted for up to 30 days (Figure 5, right graph, middle panel).

No MSP1-specific IgG ASCs were detected in the BM at day 10 of primary infection, only appearing in detectable frequencies from day 25 onwards (Figure 5, left graph, bottom panel). There was no further increase in the number of MSP1-specific IgG ASC during a secondary infection (figure 5, right graph, bottom panel).

Collectively, these data suggest that there is only a very narrow window of time following infection when malaria-specific ASCs are found in peripheral blood, and, taken with the kinetics of plasma cells in spleen and bone marrow, reflect the migration of malaria-specific plasma cells from the spleen to the bone marrow.

### CD138^+^ antibody secreting cells in peripheral blood are migrating plasmablasts

Plasma cells in peripheral blood have two potential sources; either they are newly differentiated plasmablasts migrating from the lymphoid organs to the become long-lived PC in the bone marrow (LLPC), or they could be pre-existing LLPC that have been dislodged from their bone marrow niche by the newly migrating PC [16], [39], [40]. If numbers of ASC in peripheral blood are to be used as an indication of an ongoing humoral response and development of plasma cells, then it is important that we can differentiate between these two possibilities. LLPC dislodged from bone marrow are unable to re-home to survival niches and die within two weeks in circulation [41]; it is unlikely that they contribute to ongoing humoral responses.

**Figure 6:**
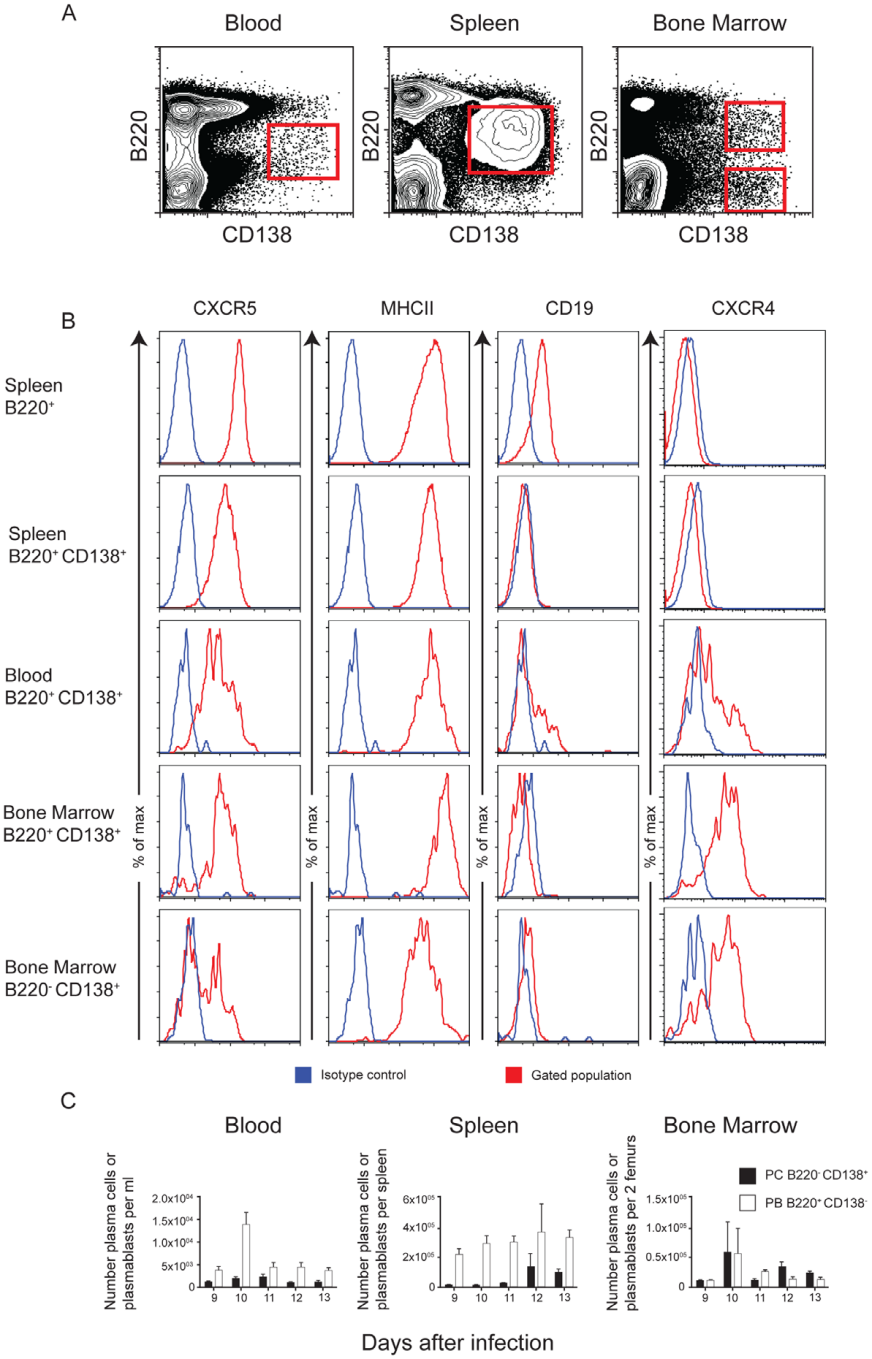
Antibody-secreting cells in peripheral blood are migrating plasmablasts. A) Gating strategy for the identification of plasmablasts and long-lived plasma cells (LLPC) in peripheral blood, spleen and bone marrow on day 10 of infection. B) Histograms showing relative expression of CXCR4, CD19, MHC class II and CXCR5, on spleen (top two panels), blood (middle panel) and bone marrow (bottom two panels). Differences between plasma cells and plasmablasts were determined by the mean fluorescence intensity of these markers on the gated populations (red histograms) showing the gradual acquisition of CXCR4 and loss of CXCR5, CD19, MHC class II on splenic B220^+^ cells (top panel), B220^+^ CD138+ cells in spleen, blood and bone marrow (middle three panels) and B220^−^ CD138^+^ LLPC in bone marrow (bottom panel). The blue histograms represent the fluorescence profile with the isotype control antibodies. C) A representative experiment out of 3 experiments showing kinetics of the appearance of plasma cells and plasmablasts during peak of infection based on their expression of B220 and CD138. The values and error bars shown are the means and the standard errors of the mean (SEM) of 5 mice.

Newly differentiated migratory plasmablasts and LLPC can be distinguished by the co-expression of the cell surface molecules B220 and CD138 (Figure 6A), in conjunction with the relative expression of other surface molecules like CD19 and MHC class II. Plasmablasts are CD138^+^ B220^+^ and express higher levels of MHC class II and CD19, whereas mature bone marrow plasma cells have downregulated CD19, B220 and MHC class II expression. In addition, the chemokine receptor CXCR4 is upregulated as B cells differentiate through plasmablasts and is maintained on bone marrow LLPC, whereas expression of CXCR5 is down regulated as B cells differentiate into plasmablasts [19], .

We assessed the expression of these molecules on the surface of plasma cell populations in blood, spleen and bone marrow defined by CD138 and B220 expression (Figure 6A). Plasma cells in peripheral blood at day 10 a primary or secondary infection. The majority of CD138^+^ PC in blood in a primary infection expressed B220 typical of plasmablasts, and thus more resembling the cells emigrating from the spleen than PC being displaced from bone marrow (Figure 6A; left panel). This was also observed during a secondary infection (data not shown). A more detailed analysis at day 10 of a primary (Figure 6B) or secondary infection (data not shown), which revealed a distinct shift in the expression of CD19, MHC class II, CXCR4 and CXCR5 between splenic B220^+^ cells and CD138^+^ cells in spleen, blood and bone marrow. CD19 was downregulated on all CD138^+^ cells compared with splenic B220+ cells, with the greatest reduction on CD138^+^ B220^−^ LLPC in bone marrow. Similarly, MHC class II and CXCR5 expression was lowest on these cells. Conversely, CXCR4 was upregulated on CD138^+^ cells compared with splenic B220^+^ cells, and was highest on bone marrow CD138^+^ B220^−^ LLPC. Thus it appears that there is a gradual acquisition or loss of the markers as B cells differentiate into LLPC. The CD138^+^ cells in blood at the peak of a primary or secondar infection were intermediate in this pathway, strongly supporting the idea that they are newly differentiated migratory plasmablasts. We therefore conclude that the transient appearance of CD138^+^ cells in the peripheral blood at the peak of infection is due to plasmablasts generated by the primary or secondary response migrating to the bone marrow niches to become LLPC. Therefore, the MSP1-specific IgG ASC response detected by ELISpot as described in Figure 5 is likely to be part of an ongoing malaria-induced immune response rather than dislodged bone marrow LLPC and in this respect sampling of blood can be used to detect a malaria-induced PC response during infection.

## Discussion

Analysis of immune responses in humans requires the use of PBMC as a source of lymphocytes and myeloid cells, which may not always reflect the ongoing or memory responses, particularly during or following infections such as malaria, when reactive cells are likely to be located in the appropriate secondary lymphoid organs [2]–[6], [45]. This is a pertinent issue when using PBMC to analyse the B cell and plasma cell responses following immunisation or infection, as B cell responses take place in lymphoid organs, and plasma cells generally reside primarily in bone marrow, but also in other lymphoid organs and inflamed tissues.

Here we have asked how far the composition of total B cells and plasma cells, and the numbers of MSP1-specific memory B cells and antibody-secreting cells in PBMC during and after a *P. chabaudi* infection in the mouse reflect those of spleen and bone marrow (the major site of induction of the B cell response and the location of long-lived plasma cells, respectively). Although the MSP1-specific B cell response may not be reflective the total B cell response to all *P. chabaudi* antigens, our data suggest that *P. chabaudi*-induced memory B cell and PC/ASC responses in blood do not consistently represent ongoing responses in the lymphoid organs, but can be reflective of the appropriate organs if the timing of sampling coincides with cell migration or trafficking.

Isotype-switched or antigen-experienced memory B cells, defined phenotypically here as CD19^+^ IgD^int^ IgM^low^, are found in peripheral blood only at low frequency following a primary and secondary infection, but are still detectable for several weeks or months afterwards. in this respect similar to the presence of isotype-switched B cells in the spleen. MSP1-specific memory B cells in blood, determined by a functional ELISpot assay, are also present at very low but detectable levels for similar periods of time, and present in increased numbers following a second infection; again with similar kinetics, but not magnitude, to the response of splenic memory B cells, and remain at persistently low but detectable levels for up to 90 days after a primary infection. These observations are in agreement with human studies, where in areas of extremely low transmission, and many years after a single exposure, *P. falciparum*-specific MBC could be detected in peripheral blood in low numbers [46]. The results from two vaccination studies are similarly encouraging, demonstrating the generation of long-lived memory B cell responses in the blood [47], [48]. It is interesting to note that the *P. falciparum*-specific MBC compartment has been observed to increase with age in children and adults, indicating that there might be a delayed acquisition of natural immunity to malaria in humans, while MBCs specific to other infections and a variety of common vaccine antigens appear to stabilise rapidly after vaccination and subsequently remain constant regardless of age [49], [50]. It is important to note that memory B cells and long-lived plasma cells represent independent arms of humoral immunity, and persistence of specific memory B cells in peripheral blood alone may not correlate with the longevity of specific humoral immunity [51].

Regarding the fate of MSP1-specific MBC during a secondary infection, the significantly higher frequency of memory B cells at day 10 of a secondary infection, followed by a reduced frequency by day 20, may reflect the differentiation of these cells into short-lived plasma cells after antigen stimulation [52], [53] as most early post-germinal centre memory B cells have a limited lifespan [14] as suggested by accompanying increase in ASC in the spleen this time of the secondary *P. chabaudi* infection. It is possible that MSP1-specific memory B cells die in the spleen as a result of a secondary *P. chabaudi* infection, as we did not observe an increase in MSP1-specific MBC in the blood or increase in plasma cells in the blood or bone marrow following the decline in MBC in the spleen after day 10 of the secondary infection. This is in line with a previous study demonstrating *P. yoelii*-induced apoptosis of pre-established MSP1-specific memory B cells in the spleen [54].

The very low numbers of specific memory B cells persisting in blood is very much in line with studies on human B memory cell responses in malaria endemic areas. For some *Plasmodium* antigens there were no, or very low intermittent memory B cells detectable [49], [55], and these are often undetectable outside of the transmission season. These findings have been interpreted as a defective or short-lived memory B cell response, but it may simply reflect the low frequencies of persisting, circulating memory B cells and the slow acquisition of malaria immunity. That there are significant numbers of specific memory B cell still present in mice in 6 weeks post infection is exemplified here by the relatively rapid increase in ASC in blood and in spleen [38] shortly after a secondary infection. It is interesting that the percentage of peripheral blood MBC which we observed in mice (less than 5% of total B cells) during primary and secondary infection is lower than that typically found in humans. This may reflect the very clean environment in which laboratory mice are housed compared with the exposure of humans to many pathogens and environmental antigens which can expand the MBC pool over time.

Many antibody reagents used to define the developmental status of B cells or plasma cells have been characterised in “steady state” or after immunisation, and not during long-lasting or repeated infection. Several groups including our own have shown that have shown that immunisation and infection with *Plasmodium* or other pathogens transiently suppress and/or alter bone marrow haematopoesis [24], [56], which is followed by an increase in export of immature cells including RAG^+^ immature B cells [57]. In agreement with these previous studies demonstrating the accumulation of RAG^+^ immature B cells in spleen and blood following administration of adjuvants or infection, we show here that during an acute primary blood-stage *P. chabaudi* infection there was an increase in the numbers of immature T1 and T2 B cells in both blood and spleen, whereas there was a transient decrease in these cells in bone marrow.

The GL7 marker is often used in mice to delineate CD19^+^ B cells that had undergone a germinal centre reaction [35], [36] and which will differentiate into plasma cells [34]. However GL7 is also expressed on early developing B cells in the bone marrow [34]. Since the numbers of immature B cells increases in blood and spleen after an acute *P. chabaudi* infection, and GL7 expression is found predominantly on T1 and T2 B cells particularly in blood, the expression of this molecule cannot be used to define B cells that have undergone maturation in germinal centres. As the presence of immature T1 and T2 B cells have also been described in human peripheral blood [58], it will be important when translating these observations to human infections to define memory or germinal-centre experienced B cells using several markers besides GL7 and using functional assays to ensure that immature B cells are not included.

The large number of immature B cells in the circulation or in the spleen can have a role in altering the outcome of the B cell response, as immature B cells are highly susceptible to tolerance [59], [60] and therefore unlikely to develop into memory cells [56]. This may be another explanation for the very low frequency of total and MSP1-specific memory B cells amongst PBMC observed by flow cytometry and ELISpot in this study and in studies on malaria-exposed humans, Here we have only measured one P. chabaudi Ag; it is also possible that the overall frequency of all malaria specific MBC and ASC is normal.

Plasma cells either defined by expression of CD138, an adhesion/growth factor receptor [61] or as MSP1-specific IgG ASC, were detected in blood at very low frequencies, and only very transiently (approximately 10 days after primary and secondary infection), in stark contrast to memory B cells, which were detectable in PBMC at all the time points sampled. [62]There was also a sharp transient increase in plasma cells in peripheral blood around day 10 of the secondary infection. In both primary and secondary infections, the MSP1-specific ASC mirrored the CD138^+^ PC in kinetics, but at substantially lower frequencies. In contrast, the dynamics of total PC in bone marrow was different; CD138^+^ PC accumulated and were present for up to 90 days (the period of observation), whereas MSP1-specific ASC were observed for up to 20 days after secondary infection (the period of observation).

Our observations fit well with previous studies demonstrating human and mouse plasma cells in blood 7 to 10 days after immunisation [17], [63], [64], and support the idea that this transient appearance of PC in blood represents the normal migration of PC after antigen stimulation and B cell differentiation. It has been previously described that most plasma cells move from their lymphoid organ to the bone marrow within one week to 10 days after B cell activation and differentiation [65], after which PC/ASC lose their ability to migrate and die [66].

It is possible that CD138^+^ cells and MSP1-specific ASC in blood may not be only plasmablasts migrating from lymphoid organ to bone marrow [16], [39], [44], but may also represent PC lost from bone marrow through ablation of the niche, or displaced through competition for the niche by new plasmablasts developing from memory B cells. Ascertaining the origin of the plasma cells detected in peripheral blood of malaria-infected humans would therefore be important in shedding light on whether plasma cell dislodgement is a phenomenon associated with malaria infection and hence another explanation for the short-lived antibody responses observed in endemic areas. Once dislodged, these plasma cells do not have the adequate potential for relocating efficiently and are thus not indicative of a developing immune response [16]. Encouragingly for human studies, the majority of CD138^+^ PC in PBMC in this *P. chabaudi* infection were newly differentiated migratory plasmablasts expressing intermediate levels of CXCR4, B220, CD19 and MHC class II [17] suggesting that they were indeed from the spleen and migrating to the BM via the peripheral blood, and thus immunologically relevant and likely to form part of the pool of long-lived plasma cells in the BM.

The results from this study of an experimental rodent malaria infection are encouraging, since they show that to some extent peripheral blood PBMC can be used to reflect malaria-induced B cell responses the spleen, particularly to detect plasmablasts destined for the long-lived survival niches in the BM. Although the exact timing of appearance of PC/ASC between mice and humans are unlikely to be the same, our study emphasises the need for proper study design when peripheral blood is used. It is important that time points selected for sample collection include the “windows” within which cells of interest are expected to be circulating in peripheral blood.

## Supporting Information

Figure S1 **Gating strategies for CD19^+^ and CD138^+^ cells in blood, spleen and bone marrow.** Single cell suspensions from mice on day 10 of a *Plasmodium chabaudi chabaudi* (AS) infection were prepared as described in Materials and Methods. The red line shows the gated regions for live cells (A), CD19^+^ and CD138^+^ cells (B) and isotype controls (C). The numbers indicate the percentage of each gated population.
(TIF)
