# Supplementary figures and images for: Distinct Kinetics of Memory B-Cell and Plasma-Cell Responses in Peripheral Blood Following a Blood-Stage Plasmodium chabaudi Infection in Mice
Source: PLoS One. 2010 Nov 23;5(11):e15007. doi: 10.1371/journal.pone.0015007 (PMC2990717; doi:10.1371/journal.pone.0015007)

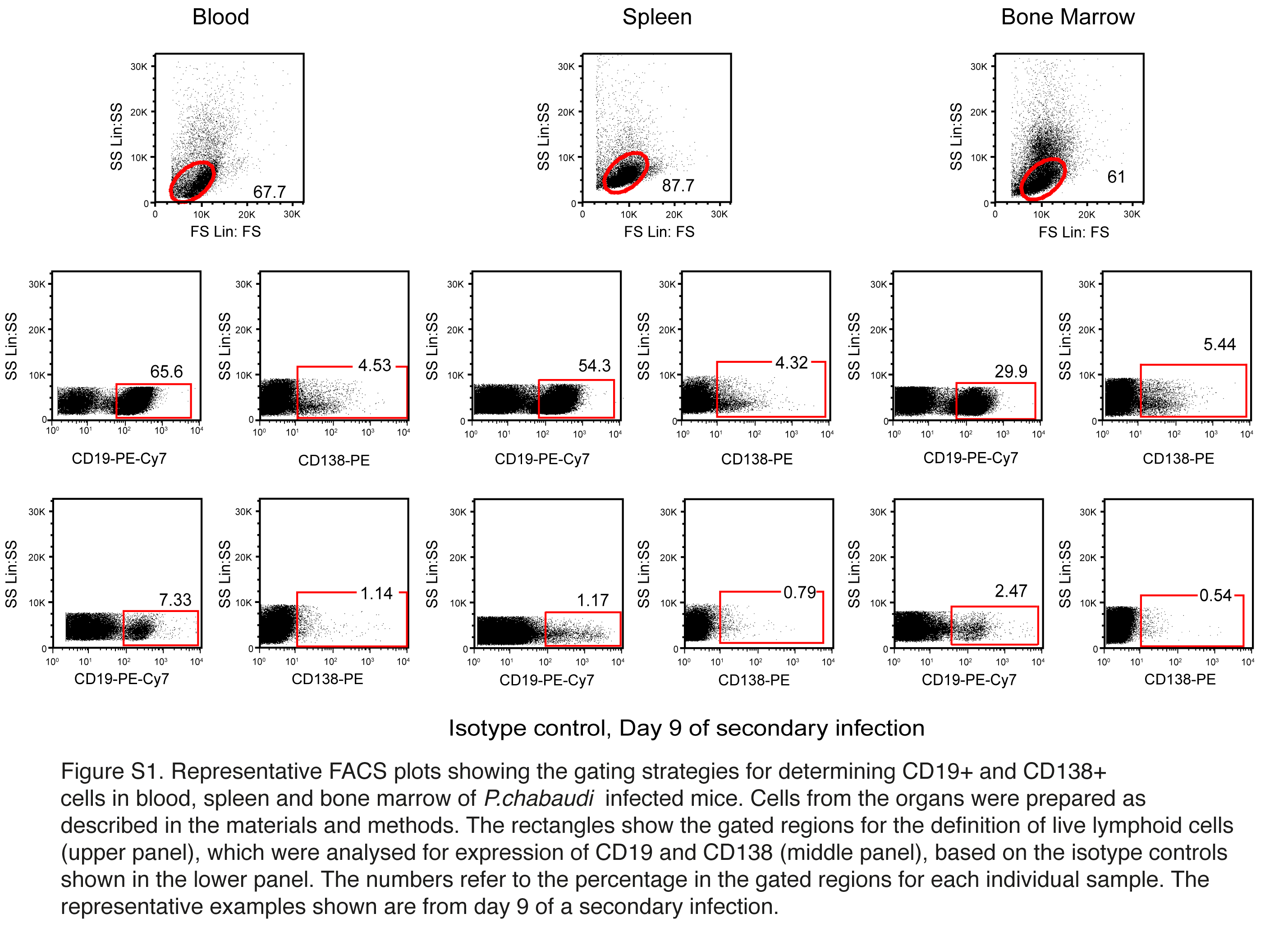

Supplement: Figure S1 — Gating strategies for CD19+ and CD138+ cells in blood, spleen and bone marrow. Single cell suspensions from mice on day 10 of a Plasmodium chabaudi chabaudi (AS) infection were prepared as described in Materials and Methods. The red line shows the gated regions for live cells (A), CD19+ and CD138+ cells (B) and isotype controls (C). The numbers indicate the percentage of each gated population. (TIF) [file pone.0015007.s001.tif]
